# Abuse and discrimination towards indigenous people in public health care facilities: experiences from rural Guatemala

**DOI:** 10.1186/s12939-016-0367-z

**Published:** 2016-05-13

**Authors:** Alejandro Cerón, Ana Lorena Ruano, Silvia Sánchez, Aiken S. Chew, Diego Díaz, Alison Hernández, Walter Flores

**Affiliations:** Center for the Study of Equity and Governance in Health Systems (CEGSS), Guatemala, Guatemala; University of Denver, Denver, CO 80208 USA

## Abstract

**Background:**

Health inequalities disproportionally affect indigenous people in Guatemala. Previous studies have noted that the disadvantageous situation of indigenous people is the result of complex and structural elements such as social exclusion, racism and discrimination. These elements need to be addressed in order to tackle the social determinants of health. This research was part of a larger participatory collaboration between Centro de Estudios para la Equidad y Gobernanza en los Servicios de Salud (CEGSS) and community based organizations aiming to implement social accountability in rural indigenous municipalities of Guatemala. Discrimination while seeking health care services in public facilities was ranked among the top three problems by communities and that should be addressed in the social accountability intervention. This study aimed to understand and categorize the episodes of discrimination as reported by indigenous communities.

**Methods:**

A participatory approach was used, involving CEGSS’s researchers and field staff and community leaders. One focus group in one rural village of 13 different municipalities was implemented. Focus groups were aimed at identifying instances of mistreatment in health care services and documenting the account of those who were affected or who witnessed them. All of the 132 obtained episodes were transcribed and scrutinized using a thematic analysis.

**Results:**

Episodes described by participants ranged from indifference to violence (psychological, symbolic, and physical), including coercion, mockery, deception and racism. Different expressions of discrimination and mistreatment associated to poverty, language barriers, gender, ethnicity and social class were narrated by participants.

**Conclusions:**

Addressing mistreatment in public health settings will involve tackling the prevalent forms of discrimination, including racism. This will likely require profound, complex and sustained interventions at the programmatic and policy levels beyond the strict realm of public health services. Future studies should assess the magnitude of the occurrence of episodes of maltreatment and racism within indigenous areas and also explore the providers’ perceptions about the problem.

## Background

Although there has been a trend of economic growth and improved health outcomes, Latin America continues to be a region characterized by deep-rooted inequalities where indigenous groups bear a disproportionate burden of poverty, ill health and preventable mortality, despite accounting for only about 8 % of the total population [1]. Disparities between indigenous and non-indigenous groups are compounded by widespread social exclusion and discrimination. This is manifested in denial of rights, racist and prejudicial treatment, and limited access to quality public services [2]. The systematic marginalization indigenous people face in the region reflects long-standing historical and social processes of dispossession of land, labor exploitation and exclusion from political participation [3].

Guatemala has one of the highest proportions of indigenous groups in Latin America, and approximately 45 % of the population belongs to one of the 23 ethnic groups. About 95 % of the indigenous people identify as Maya, the main victims of the internal civil conflict that engulfed the country in the second half of the 20th century. It is estimated that 83 % of the two million victims of the war belonged to one of the different Maya groups, who suffered geographical displacement and were the systematic target of rape, torture, and mass killings [4, 5]. Two decades have passed since the signing of the 1996 Peace Accords, and despite the call for equitable social and economic development for all Guatemalans, Mayans continue to face barriers to participate in public decision-making, including politics. These ethnic groups are underrepresented in government, industry and in almost every occupation except subsistence farming [6, 7]. Limited access to formal education contributes to lack of opportunities, and limited social mobility communication for indigenous Guatemalans, as many of them are not able to communicate effectively in the country’s official language- Spanish [7]. These intersecting social determinants, together with isolation from political and economic life, as well as communication barriers, contribute to the stark levels of inequities in the health of Guatemalans, where indigenous women are more than twice as likely to die in childbirth than their non-indigenous counterparts (with maternal mortality ratios of 163 and 78, respectively) and overall, the life expectancy of indigenous people is 13 years less than that of non-indigenous Guatemalans [8].

In this context, strategic efforts to provide Universal Health Coverage (UHC) acquire the moral imperative to improve equity, with specific focus on meeting the needs of indigenous population groups [9]. In Latin America, health systems tend to be structurally segmented, with private, social security and public sub-systems providing services for different population groups. Public healthcare services are the main source of care of the indigenous population in Guatemala. However, public facilities are chronically underfunded, have health worker shortages, regular stock-outs of medicines and supplies, and inadequate infrastructure [10, 11].

Improving health equity levels requires removing the multiple barriers indigenous populations encounter when seeking care, and tackling such barriers has been a central focus of Latin American UHC strategies. Despite such efforts, studies show that cultural barriers continue to limit indigenous people’s access to quality care. Research done in Bolivia, Nicaragua and Guatemala has documented the mistreatment and abuse indigenous women experience during child delivery, highlighteding how such treatment negatively influences patients’ willingness to utilize health services [12–14]. Indigenous men and women have reported differential treatment and provisions of services based on ethnicity in Mexico and Peru. They describe being shamed by providers who treat them as ignorant or inferior, and being denied care [15, 16]. Language barriers have also been highlighted as an important constraint to receiving, or even seeking healthcare [17, 18]. Understanding the discrimination and abuse indigenous people experience may lead to a reduction of the cultural barriers to UHC, and can help to create a health system able to address the needs and expectations of vulnerable and marginalized groups [3]. Our aim in this study, was to explore whether indigenous patients and their family perceive abuse and discrimination when accessing public healthcare services in rural Guatemala.

## Methods

### Setting: the highlands of Guatemala

The western highlands of Guatemala, which have the highest levels of poverty and illiteracy in the country, are predominately populated by Maya indigenous groups. The majority of the population lives in small rural communities with limited access to public services, including health, education, sanitation, and transportation infrastructure. Although the Ministry of Health (MoH) has the legal obligation to provide services from the first level (community) up to tertiary care, public health service are routinely understaffed and work with limited, low quality resources. Access is further complicated by physical and financial barriers, because although care is free, including medicines-when available, travel expenses to reach health facilities, loss of work, and the cost of medicines often represent considerable expenditures for a Maya family [19]. Community-level coverage mainly consists of health posts run by an auxiliary nurse, and they often lack the medical supplies needed to provide care [19]. This coverage can be supplemented by Non-Governmental Organizations (NGOs) contracted by the MoH to render services through mobile health teams, who travel to remote communities to provide a specific package of services targeting maternal and child health. Within the package of services provided by contracted NGOs, there is no provision of care for children older than five, men of any age, or women past their reproductive years [20, 21].

### Data collection

Fourteen municipalities from the western highlands of the country were purposively selected for this study [22]. These municipalities are part of a larger project promoting social accountability of health care facilities in rural areas, and their work with health services monitoring led to the identification of the problem of discrimination and abuse. An overview of the sample municipalities and study participants can be found in Table 1.

**Table 1:** Description of the sample municipalities

*Region*	*Municipality*	*Participants*	
		*Men*	*Women*
Huehuetenango	Concepcion Tutuapa	9	6
	Ixtahuacan	16	16
	Ixchiguan	0	12
	Cuilco	8	8
	Tectitan	8	5
Alta Verapaz	Carcha	5	13
	Chisec	6	8
	Lachua	2	13
Quiche	Uspantan	2	10
	Nebaj	6	9
Totonicapan	Totonicapan	0	8
	La Reforma	2	9
Solola	Nahuala	5	4

The first step in the data collection process was to visit each of the selected villages and present the aims for this study. The second step was to organize focus group discussions (FGDs), which were arranged with the help of community leaders. The goal of the FGDs was to identify episodes of mistreatment. The number of episodes identified in each municipality can be found in Table 2. A total of 13 FGDs where carried out by SS and ASC. When relevant, a social sciences-trained interpreter from indigenous languages to Spanish mediated conversations. Interviews were later translated and transcribed into Spanish. Because of the low levels of literacy in the selected villages, the research team sought verbal consent from all the participants, who were informed they could leave the discussion at any point, and that doing so would carry no negative repercussions.

**Table 2:** Type of episode of mistreatment by municipality

*Region*	*Municipality*	*Episodes of mistreatment*	*Discrimination*	*Abusive treatment*	*Neglect of professional ethics*
Huehuetenango	Concepcion Tutuapa	10	6	4	0
	Ixtahuacan	7	0	4	3
	Ixchiguan	9	1	4	4
	Cuilco	7	0	4	3
	Tectitan	23	5	11	7
Alta Verapaz	Carcha	8	2	2	4
	Chisec	11	3	4	4
	Lachua	11	4	4	3
Quiche	Uspantan	7	2	3	2
	Nebaj	8	2	2	4
Totonicapan	Totonicapan	8	2	2	4
	La Reforma	16	5	7	4
Solola	Nahuala	7	2	4	1

### Data analysis

Thematic analysis is an analytical method used to search through qualitative data with the aim of finding recurrent patterns that can be grouped into themes through the use of discursive interpretation [23, 24]. The first step in this process involved the careful reading of the transcriptions from the fieldwork, with the aim of having the research team be familiar with the data. Afterwards, AC, SS, ASC and DD did an initial round of coding. These were later discussed and a coding scheme was defined. A first organization of themes was proposed by AC, SS, ASC and DD, and these were later revised and refined by ALR, AH and WF. Finally, AC, AH and ALR named the themes and AC, AH, ALR and WF narrated the presented version of the themes and selected quotes.

### Ethical considerations

In Guatemala it is only necessary to ask for clearance from an ethical board when conducting clinical trials or carrying out tests on humans. However, we additionally sought ethical clearance by informing community leaders and other authorities about the project and its purpose. We presented our methodology to the participants, and secured verbal informed consent. All participants knew there would be no penalties or any type of consequences if they chose to withdraw or not participate in our study. We asked permission to record the interviews, or to take notes, and guaranteed anonymity. In the findings we do not present any names of people or villages, choosing to only identify regions. Finally, we have shared the results of our study with the participants.

## Results

Three themes were identified through the analysis of the episodes of mistreatment documented in this study. Episodes narrated and discussed by participants pointed to discrimination in access, abusive treatment, and neglect of professional ethics in health care services. Table 2 presents the number of episodes that talked primarily about each of these themes, which are described in the following sections.

### Theme 1: Discrimination in access to care

For indigenous people trying to access care in a public health facility, the ability to speak Spanish is a major determinant. Without a good command of the language, patients will not be able to explain their symptoms or to follow conversations laden with medical terms. Participants reported that physicians and nurses might deny care to patients that did not speak Spanish. Healthcare providers would not seek out any interpreters; even when family members were present and willing to help. This led some participants to believe that speaking a Maya language put people in disadvantage, and that only Spanish native speakers will receive the care they seek. One woman reported:

*‘When I went to the hospital, I could not explain to the doctors how I felt because I do not speak Spanish very well. The doctors did not understand what I wanted to tell them, and they did not let any of my relatives come in* [to the examination room to help me] *and explain to them how I felt’.* Woman from Solola.

The Guatemalan Ministry of Health has an explicit policy of prioritizing the delivery of maternal and child health services. Although services to other population groups are provided in larger urban facilities, in rural facilites with limited resources mostly target expectant mothers, women who want to avoid pregnancy, and young children. Services for any other age or gender group are usually not provided. People that do not belong to any of these age-groups do not feel welcomed at the facilities. Participants reported that when men go to a health care facility, they get turned away with the explanation that ‘men never really get sick’ and that the resources that exist are only there for women. It was reported that in Huehuetenango, single women and girls that seek healthcare are asked if they are pregnant. If they say no, they are sent away.

In the experience of many of the participants in our study, these barriers can only be overcome through having personal connections to the staff at the health facility. Influential patients and families, routinely receive better attention, have shorter waiting times and are given medicine at the end of the visit. A patient without these connections would routinely be made to wait, or be told that there are no drugs in stock.

*‘One day my daughter had an infection in her foot, so I took her to the hospital. I got there and asked if they had medicine for my daughter. ‘Sit over there, I’ll go check’ the doctor told me, and he was angry. Then a lady came and she got care while I waited outside and my daughter was still there. Then another lady came and she also got care pretty quickly. The nurse would say ‘you stay there waiting’ and I would tell them my daughter was very sick. Five people came after us and got care. When they finally saw me they only half-checked my daughter’s foot and we didn’t get any medicine. The other people did get medicine, while we only got a prescription. They only care for the people they know, while I came from far away and the doctor doesn’t care…’* Man from Alta Verapaz

### Theme 2: Abusive treatment in care

The cases of abuse we documented ranged from lying to patients and their families, to forcing unwanted and painful procedures on individuals. In many cases, the abuse involved several types of mistreatment. The most commonly reported form of abuse was yelling, which often occurred in combination with other types of mistreatment. According to the participants, belonging to an indigenous ethnic group and being poor were the motivation behind the abusive treatment they receive. Physicians and nurses would yell out disrespectful remarks along with commands or orders. Many of the participants felt powerless after being yelled at by a health care provider. Others added that, in some situations, they were in urgent need of care and felt that they could not defend themselves, so they had to put up with the abuse. One woman told us:

*‘It happened to me when I was pregnant. I was in the hospital bed and I had strong pains, so I was moving a lot and screaming. The doctor shouted at me and said ‘do not yell and hold it in. Were you screaming this way when you were making this baby? No, you were going about it slowly and smoothly, right? So now put up with this.*’ Woman from Huehuetenango

It was reported that patients and families are frequently lied to during interactions with the staff from health facilities. A patient might be asked to wait for hours, only to later be told that the clinical staff was not in that day. Other times they would be told that a certain diagnostic test could only be done at private laboratories, or that there were no drugs at the health facility. In some severe cases, patients were not told the truth about the gravity of their situation, or were never told their diagnoses. The effects of these lies can range from feelings of desperation or confusion to economic loss through extra out-of-pocket expenditures. One woman recounted:

*‘My son had a stomachache and I took him to go get checked, and it turned out his liver was sick.* [the doctor] *sent us to get blood, feces and urine tests to a private facility because the results come back faster there… I had to pay for the tests, and then went back to the health center with the results. The nurse then told me the doctor was not there, he had gone home for lunch at noon and would not return until 4 pm. I was left there waiting. I was hungry because I used the money [60USD] I had borrowed to pay for the tests… meanwhile the doctor is having lunch at his house. I was there waiting and going back and forth while the nurses were just laughing and not paying any attention to me*’. Woman from Totonicapan.

Participants reported that it is common practice for patients to be forced to accept procedures without being properly informed beforehand by the staff in the health facility. In the case of institutional deliveries, women are often forced to undress, made to take cold baths, and shaved without consent. Sometimes, procedures like caesarean sections and even sterilizations are performed without informing the expectant mother or the rest of the family:

*‘One day, we traveled to the hospital because my wife was going to deliver, but we had to wait for a long time before someone finally saw her. Then they took her in and very quickly performed a C-section. They did not examine her beforehand… I asked the doctor why he had done this and he said that my wife was in too much pain, and that this was why they also injected her with something to stop her from having more children. I got very upset because they should have asked…’* Man from Solola

It is not only expectant mothers that are forced into procedures without any explanation:

*I had a stomachache and could not stand the pain. I went to the hospital and they did not have any medicines to give me, so the doctors… washed my stomach out with cold water. They were very unkind. They forced me to do it. I did not want to…’* Woman from Solola

### Theme 3: Neglect of professional ethics

In general, participants thought that having the vocation to become a physician or a nurse was a gift that came with specific duties, and being a healthcare professional meant they had a calling to serve the community. These professions, they expressed, come with a specific set of duties and with a code of ethics that should translate into kindness and polite treatment to patients.

The care that physicians and nurses provide should be personalized, and healthcare professionals should take the time to explain to patients about their illnesses and explain the treatment patiently. This would make people feel cared for and like they were in a place where they could feel better. However, this type of treatment is scarce, as one man recounted:

*‘Never did the doctor or the nurse sit down with me and explain what medicine I should buy. They talk to you in a hurry; they just say there is no medicine. The doctor did not even smile at me. They only give you a prescription and they do this while they look somewhere else. They never even look at your face’.* Man from Alta Verapaz.

According to the participants, it is not acceptable for physicians and nurses to leave patients alone in their beds, or not to be present to help them take their medicine. Patients wished the health staff would show more interest in how patients feel, as well as have more compassion and empathy. In stark comparison, traditional birth attendants are perceived as kind and as living up to what our participants described as ‘their professional code of conduct’.

As a way to improve the quality of care given out at health facilities, participants expressed the need for healthcare providers to be better supervised. This would lead to more accountability and an improvement in their demeanor and attitudes.

## Discussion

The experience of discrimination, mistreatment while seeking healthcare is not exclusive to the Guatemalan setting. Non-consented care, neglect, and verbal and physical abuse are among the most commonly reported complaints in rural areas of Africa, where about a third of women report at least one form of disrespect when delivering a child in a public facility [25–27]. Qualitative studies show that discrimination and abuse play a prominent role in women’s experience of institutional childbirth [27]. Women in Guatemala and in Ghana have highlighted the humiliation and suffering experienced as a result of verbal and physical abuse. For them, the treatment they received was understood as the way through which providers punish them for their culture and ethnicity [3, 28, 29]. In addition, the abuse makes women feel ashamed of their poverty, their way of dressing, hygiene and their use of traditional medicine [29].

Although the body of literature documenting discrimination, mistreatment and abuse of patients continues to grow, it is limited by its focus on care during childbirth Our study contributes to the expansion of barriers that keep patients from seeking care as a broader phenomenon that affects access to quality care for men, children and women that seek non-obstetric care. As Universal Health Coverage continues to gain steam in global health discussions, particularly those around the Sustainable Development Goals [9, 30], so must our understanding of the different barriers that keep patients and families from accessing the care they need. Indeed, without a deep understanding of mistreatment and abuse, we may repeat the same mistakes already identified in the reductionist, indicator-centric Millennium Development Goals [31, 32].

Our study also contributes to the body of knowledge that shows that discrimination plays a prominent role in the experiences that indigenous people have when accessing healthcare services [1, 2, 33]. The experiences expressed by the participants in our study provide insight into how discriminatory practices can restrict access to quality healthcare, of the verbal, psychological and physical abuse experienced, as well as the neglect of professional ethics in providers’ treatment of patients. In Guatemala, where most healthcare providers belong to the dominant, non-indigenous ethnic group, experiences of discrimination are framed in the broader social exclusion situation that support the perceived inferiority of the indigenous people, based on their ethnicity, educational level, and socioeconomic status [3, 5, 34]. In addition, the power differentials that exist between patient and provider contribute to the chasm of social distance between providers who earn a salary and are educated, and patients, who are poor and illiterate or with limited formal education [29].

A previous study found that rural, indigenous patients felt that the mistreatment they receive within the healthcare system is systemic and reflects long-standing racial issues [3]. When these abuses are shared at the community level, it shapes the environment in which future health seeking decisions are made, and contributes to feelings of distrust of the health system based on ethnicity [35–40].

Although all authors strived to present a holistic view of the phenomenon of discrimination of indigenous patients based on the instances described, we would like to present the limitations to our study. We used qualitative data collection methods with predominantly indigenous population in rural Guatemala. Our findings can not be generalizable. Further quantitative studies could provide insight into the prevalence of experiences of mistreatment, however, such household survey techniques should be approached with caution due to the sensitive nature of the issue. During our fieldwork we only sought to collect the views of patients and their families, and we did not interview any service provider about their views on this issue. Further studies are needed to understand the health provider-side of this issue, and we are planning a second study that will explore the perception of providers in order to offer a more comprehensive view of the phenomenon of mistreatment and to identify contextually-relevant strategies for promoting respectful care that is accessible to all.

## Conclusion

This study has documented episodes in which indigenous people using public health care services experience discrimination and abuse from providers. In order to promote respectful and dignified care within the Guatemalan health system, a human rights-based approach to UHC can provide a basis for developing a more inclusive and equitable health system for the entire population. Through the use of human rights as the normative base for health policies, practices of abuse and discrimination as the ones described in this study, would be seen as violations to basic rights of freedom from violence and discrimination. In addition, it is relevant to further explore and understand the structural determinants within society and public policies, that contribute to the existence of discrimination and abuse in health and other public services.
